# EBV reactivation as a target of luteolin to repress NPC tumorigenesis

**DOI:** 10.18632/oncotarget.7967

**Published:** 2016-03-08

**Authors:** Chung-Chun Wu, Chih-Yeu Fang, Hui-Yu Hsu, Hsin-Ying Chuang, Yu-Jhen Cheng, Yen-Ju Chen, Sheng-Ping Chou, Sheng-Yen Huang, Su-Fang Lin, Yao Chang, Ching-Hwa Tsai, Jen-Yang Chen

**Affiliations:** ^1^ National Institute of Cancer Research, National Health Research Institutes, Zhunan, Taiwan; ^2^ National Institute of Infectious Diseases and Vaccinology, National Health Research Institutes, Tainan, Taiwan; ^3^ Department of Microbiology, College of Medicine, National Taiwan University, Taipei, Taiwan; ^4^ Department of Pathology, Wan Fang Hospital, Taipei Medical University, Taipei, Taiwan

**Keywords:** nasopharyngeal carcinoma, relapse, Epstein-Barr virus, reactivation, luteolin

## Abstract

Nasopharyngeal carcinoma (NPC) is a malignancy derived from the epithelial cells of the nasopharynx. Although a combination of radiotherapy with chemotherapy is effective for therapy, relapse and metastasis after remission remain major causes of mortality. Epstein-Barr virus (EBV) is believed to be one of causes of NPC development. We demonstrated previously that EBV reactivation is important for the carcinogenesis of NPC. We sought, therefore, to determine whether EBV reactivation can be a target for retardation of relapse of NPC. After screening, we found luteolin is able to inhibit EBV reactivation. It inhibited EBV lytic protein expression and repressed the promoter activities of two major immediate-early genes, Zta and Rta. Furthermore, luteolin was shown to reduce genomic instability induced by recurrent EBV reactivation in NPC cells. EBV reactivation-induced NPC cell proliferation and migration, as well as matrigel invasiveness, were also repressed by luteolin treatment. Tumorigenicity in mice, induced by EBV reactivation, was decreased profoundly following luteolin administration. Together, these results suggest that inhibition of EBV reactivation is a novel approach to prevent the relapse of NPC.

## INTRODUCTION

Nasopharyngeal carcinoma (NPC) is a malignancy derived from the epithelial cells of the post-nasal cavity and has a unique geographic and ethnic distribution. Although rare worldwide, it is prevalent in areas such as southern China, Southeast Asia, northeast India, North Africa and among the native population of Canada and Alaska. There are an estimated 80,000 new cases per year (0.7% of all cancers), less than 1 per 100,000 globally [1]. The 5 years survival rate is 60%; treatment at early stages of disease leads to a good 5-year survival rate (80-95%) but not for late stage disease (40-50%). NPC is markedly radiosensitive and radiotherapy is the primary mode of treatment; however, chemoradiotherapy has been shown to be superior to radiotherapy alone for advanced NPC patients [2]. Recently, with the improvement in combination of radiotherapy provided by neoadjuvant chemotherapy, the survival rate has increased significantly [3–5]. However, relapse and metastasis after remission remain major causes of mortality. Prevention of relapse and metastasis seems to be the most important issue in the study of NPC.

EBV, a member of the herpesviruses, has a linear double-stranded DNA genome of around 170 kb. The replication cycle includes latent and lytic stages and the switch from latency to the lytic cycle is known as reactivation [6, 7]. EBNAs 1, 2, 3A, 3B, 3C and LP and LMPs 1, 2A and 2B are expressed during latency. Upon reactivation to the lytic cycle, the immediate early genes Zta and Rta are expressed first, followed by the early genes (DNase, DNA polymerase, thymidine kinase, etc.) and late genes (VCA and MA, etc) [8]. Elevation of antibodies against EBV lytic gene products has been considered as a marker of EBV reactivation *in vivo* [9–11]. The infection is ubiquitous in most human populations, with no obvious symptoms. This virus has been shown to be the etiological agent of infectious mononucleosis and is associated with many human malignancies, including African Burkitt's lymphoma and NPC [8].

EBV infection, consumption of nitroso-compounds and genetic factors are considered to play important roles in the carcinogenesis of NPC [12, 13]. Elevation of antibodies against EBV in NPC patients and the presence of the EBV genome and expression of EBV genes in NPC tissues indicate the close association of EBV infection with NPC [14–20]. Individuals with higher levels of antibodies against EBV tend to have a high risk of NPC development [19]. Recent epidemiological studies indicated that fluctuation of antibodies to EBV occurs prior to the onset of NPC [21, 22]. These results suggest that EBV may contribute to the initiation of NPC. To elucidate the role of EBV in the initiation of NPC, a model system of EBV infection and reactivation in normal nasopharyngeal epithelial cells is required urgently. Unfortunately, there is no such model system available at this time.

Through years of studies, it was proposed that latent EBV infection contributes to the development of NPC after the high grade pre-invasive dysplasia [23]. Among the EBV latent proteins, latent membrane protein 1 (LMP1) is considered to make the most significant contribution to the development of NPC. In addition to the induction of genome instability [24–27], it has been shown that LMP1 induces matrix metalloproteinase 1 to increase metastasis, and interleukin-8 to increase angiogenesis, of NPC [28–30]. One of the most interesting features is that LMP1 induces hypoxia-inducible factor 1α (HIF1-α) and this subsequently contributes to the increased expression of vascular endothelial growth factor (VEGF) [31]. Further study indicated the up-regulation of HIF1α is through Siah1 to down-regulate prolyl hydroxylases 1 and 3 [32]. More strikingly, LMP1 was found to promote NPC progression through increased levels of HIF1α in the exosomes of NPC cells [33]. The pathogenic role of LMP1 in NPC has been reviewed recently [34].

In our laboratory, we have established the EBV-positive NPC cell lines, NA and HA [35] from the EBV-negative NPC line TW01, derived from an NPC patient in Taiwan [36]. Because most NPC can be treated with remission by radio-chemotherapy, NA, HA and TW01 cells are considered as residual EBV-positive and –negative NPC cells after remission and may be informative regarding the relapse of NPC. Using these cells as a model system, we could investigate the role of EBV infection in the carcinogenesis of NPC cells.

Genomic instability is one of the hallmarks of cancer [37]. We found that recurrent EBV reactivation contributes much more profoundly than latent infection to the genomic instability and tumorigenesis of NPC cells [38]. We demonstrated further that the expression of EBV lytic genes contributes to the genomic instability of NPC cells [39–41]. In particular, recurrent expression of BALF3, a homologue of terminase, does not induce cytotoxicity but mediates genomic instability and progressive malignancy [41]. These results suggest the importance of lytic infection, probably abortive, for the relapse of NPC. We therefore asked whether EBV reactivation can be a target for the prevention or retardation of relapse of NPC.

Recently the “nutraceutical” concept has become prominent. Scientific evidence has shown that vegetables and fruits contain phytochemicals, such as polyphenols, terpenes and alkaloids, that may provide substantial health benefits, other than basic nutrition [42]. Epidemiological studies indicate that populations that consume foods rich in vegetables and fruits have a lower incidence of cancers [43]. Lycopenes from tomatoes and vitamin D have been shown to be useful for the treatment of prostate cancers [44–46]. Histone deacetylase (HDAC) inhibitors are also considered as potential cancer therapeutic agents and some are the subjects of clinical trials [47]. In a region of China with a high-risk for NPC, inhabitants living in a particular area with a low NPC prevalence have a lower potential for endogenous nitrosation, suggesting the presence of nitrosation inhibitors in their diet [48]. It is worthwhile to seek more and better natural products for NPC prevention. These results led us to look for natural compounds which may prevent EBV reactivation in NPC cells in order to find dietary supplements which may be useful for the prevention of EBV reactivation and subsequent NPC relapse after remission.

After extensive screening, we focused on luteolin, a member of a group of dietary flavonoids found abundantly in medicinal herbs, fruits and vegetables (e.g. parsley, green peppers, citrus, celery and chamomile). Luteolin is known to be a good free radical scavenger and inducer of tumor apoptosis [49] and also has valuable effects in cancer prevention and therapies [50]. It has been reported to have good effects in anti-angiogenesis, anti-metastasis, anti-inflammation and estrogenic regulation, and to regulate many signaling pathways [51, 52]. However, although a few studies have been published, the question whether luteolin has anti-viral activity is less well understood [53, 54].

In this study, using the NPC cell lines NA and HA, luteolin is shown to inhibit EBV reactivation significantly by suppressing the promoter activities of two immediate-early genes, Zta and Rta. Furthermore, we found that luteolin treatment not only suppressed EBV reactivation but also reduced genomic instability and repressed the tumorigenic features induced by repeated EBV reactivation *in vitro* and *in vivo*, suggesting that inhibition of EBV reactivation is a novel target to overcome the relapse of NPC.

## RESULTS

### The cytotoxicity of luteolin to NPC cells, determined by WST-1 assay

**Figure 1:**
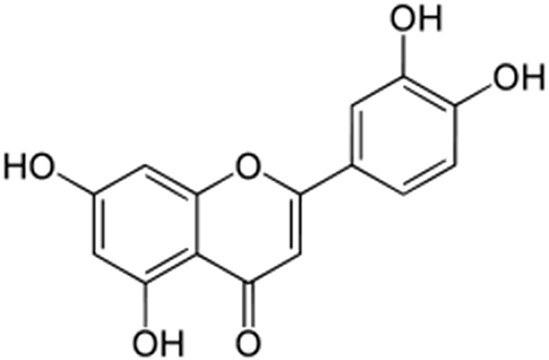
The chemical structure of luteolin

Luteolin is a flavone with a classic flavonoid 2-phenylchromene-4-one ring structure (Figure 1). NA and another EBV-infected NPC cell line, HA, were used to address the question whether luteolin can inhibit EBV lytic reactivation. First, we sought to determine the cytotoxicity of luteolin to NA, HA and their parental TW01 and HONE-1 cells, to rule out any effect of cellular toxicity. Each cell line was cultured in 96-well plates and, after plating, various concentrations of luteolin were administered for 24 hr or 48 hr to determine its cytotoxicity by WST-1 assay. The values of half maximum of cytotoxicity concentration 50 (CC_50_) for 48 hr treatment of NA and TW01 were 124 and 115 μM, while the values for HA and HONE-1 were 78 μM and 68 μM (data not shown). Taken together, we determined that the CC_50_ value of luteolin was 68 to 272 μM for 24 and 48 hrs, which is similar to that in other epithelial cancer cells [54, 55]. Thus we chose 1∼50 μM as our working concentrations for further studies.

### Luteolin inhibits the expression of EBV lytic proteins

**Figure 2:**
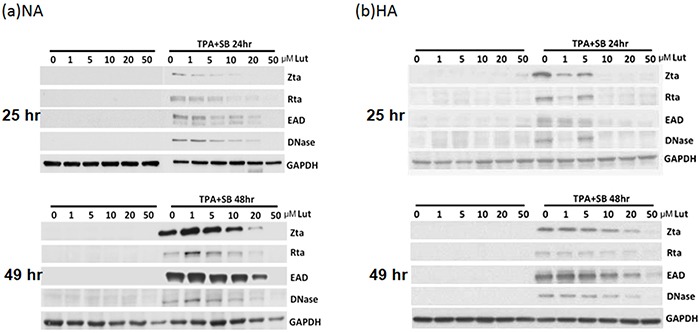
Luteolin inhibits expression of EBV lytic proteins in EBV-positive cells Epithelial cells NA **a.** and HA **b.** were subjected to western blotting. Various concentrations of luteolin were added to the cells for 25 hr to detect enhancement of reactivation. Cell lysates were collected for western blotting. For detection of inhibition of reactivation, the cells were pre-treated with various concentrations of luteolin for 1 hr, then TPA (40 ng/ml) and SB (3 mM) co-treatment was used for EBV induction. After 24 and 48 hr of incubation, cell lysates were analyzed by western blotting with antibodies against EBV Zta, Rta, EAD, DNase and GAPDH.

After induction or stimulation, the first lytic EBV proteins to be expressed are the immediate early proteins, Zta and Rta, followed by numerous early and late proteins, and subsequently the release of infectious virions. To investigate whether luteolin induces EBV into the lytic cycle, NA cells were plated for 24 hr and then the cells were treated with various concentrations of luteolin. After 25 or 49 hr, cell extracts were harvested for western analysis. As expected, luteolin did not induce expression of the lytic proteins Zta, Rta, EAD and DNase after treatment for 25 and 49 hr, shown in the left panels of Figure 2a, suggesting that it could not induce the EBV lytic cycle in NA cells. To avoid the possibility of cell specificity, another EBV-positive cell line, HA, was examined using the same procedure and with a similar result: luteolin treatment also did not induce EBV reactivation in HA cells (left panels, Figure 2b). Next, we tried to determine whether luteolin can block EBV reactivation from latency. For NA and HA cells, TPA+SB (TS) treatment is a common inducer of EBV lytic reactivation and can activate EBV effectively and induce significant expression of lytic proteins [35]. Moreover, detection of lytic protein expression is a sensitive method for evaluating EBV reactivation [35]. In order to ensure more effective luteolin treatment, NA and HA cells were pre-incubated with various concentrations of luteolin for 1 hr prior to treatment with TPA (40 ng/ml) and SB (3 mM). After further incubation for 24 or 48 hr, cell extracts were collected for the detection of lytic proteins by western blot analysis. As shown in the right-hand panels of Figure 2a, TPA+SB treatment induced the expression of the EBV lytic proteins Zta, Rta, EAD and DNase without luteolin treatment. Meanwhile, the expression of EBV lytic proteins was reduced slightly following treatment with 1 and 5 μM luteolin for 24 and 48 hr induction, and decreased significantly with 5 μM luteolin for 24 hr and 10 μM for 48 hr induction. Lytic protein expression was undetectable following treatment with 20 and 50 μM luteolin for 24 and 48 hr after induction (Figure 2a). In HA cells, luteolin inhibited lytic protein expression significantly with 10 μM treatment for 24 hr and 50 μM for 48 hr, which were higher concentrations than used for the treatment of NA cells (right panels, Figure 2b). Luteolin inhibited all detectable EBV lytic protein expression completely at 50 μM, which was similar to the treatment of NA cells (right panels, Figure 2b). Similarly, luteolin had an inhibitory effect on the expression of EBV lytic proteins after induction in C666-1 cells (Supplementary Figure S1a). Taken together, these results suggest that the flavonoid luteolin cannot induce EBV into the lytic cycle but, rather, inhibited EBV entry into the lytic cycle in EBV-positive cells.

### Luteolin decreases the populations of EAD-expressing cells, monitored by flow cytometric analysis

**Figure 3:**
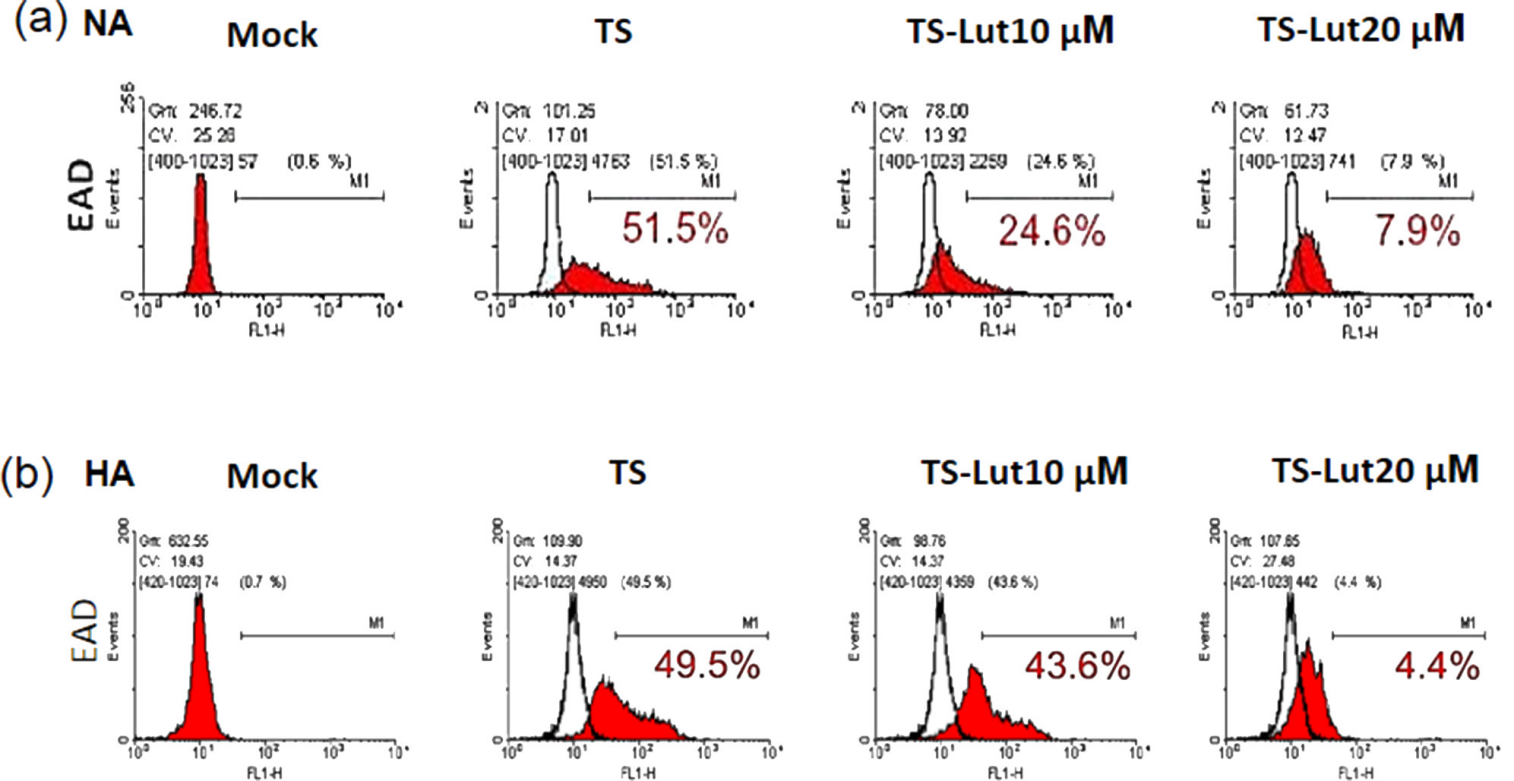
Luteolin decreases the populations of EAD-expressing cells NA **a.** and HA **b.** cells were processed for flow cytometric analysis. For detection of reactivation inhibition, the cells were pre-treated with various concentrations of luteolin for 1 hr, then TPA (40 ng/ml) and SB (3 mM) were added for EBV induction. After 24 hr of incubation, the cells were analyzed by flow cytometry with antibody against EBV EAD. TS: TPA+SB; TS-Lut10 μM: TPA+SB+luteolin (10 μM); TS-Lut20 μM: TPA+SB+luteolin (20 μM).

Next, we examined the inhibition of EBV lytic reactivation by luteolin and detected EAD expression in NA and HA cells using flow cytometric analysis after luteolin treatment, with and without TS induction. NA and HA cells were pre-treated with various concentrations of luteolin for 1 hr, then co-treated with TPA (40 ng/ml)/SB (3 mM). After 48 hr induction, the cells were collected for detection of EAD by flow cytometric analysis. The percentage of EAD expressing cells was estimated to determine the amount of EBV lytic reactivation. For NA cells with 48 hr induction by TS, the population of EAD-positive cells was 52% without luteolin treatment, while it was 25% and 8% after treatment with 10 μM and 20 μM luteolin, respectively (Figure 3a). Meanwhile, 50% of HA cells expressed EAD after TS induction without luteolin treatment (Figure 3b), lower than NA cells (52%). The percentages of HA cells expressing EAD were 44% and 4% following treatment with10 and 20 μM luteolin, respectively (Figure 3b). These results provide further evidence that luteolin can inhibit the EBV lytic cycle.

### Luteolin represses the transcriptional activities of Zp and Rp

**Figure 4:**
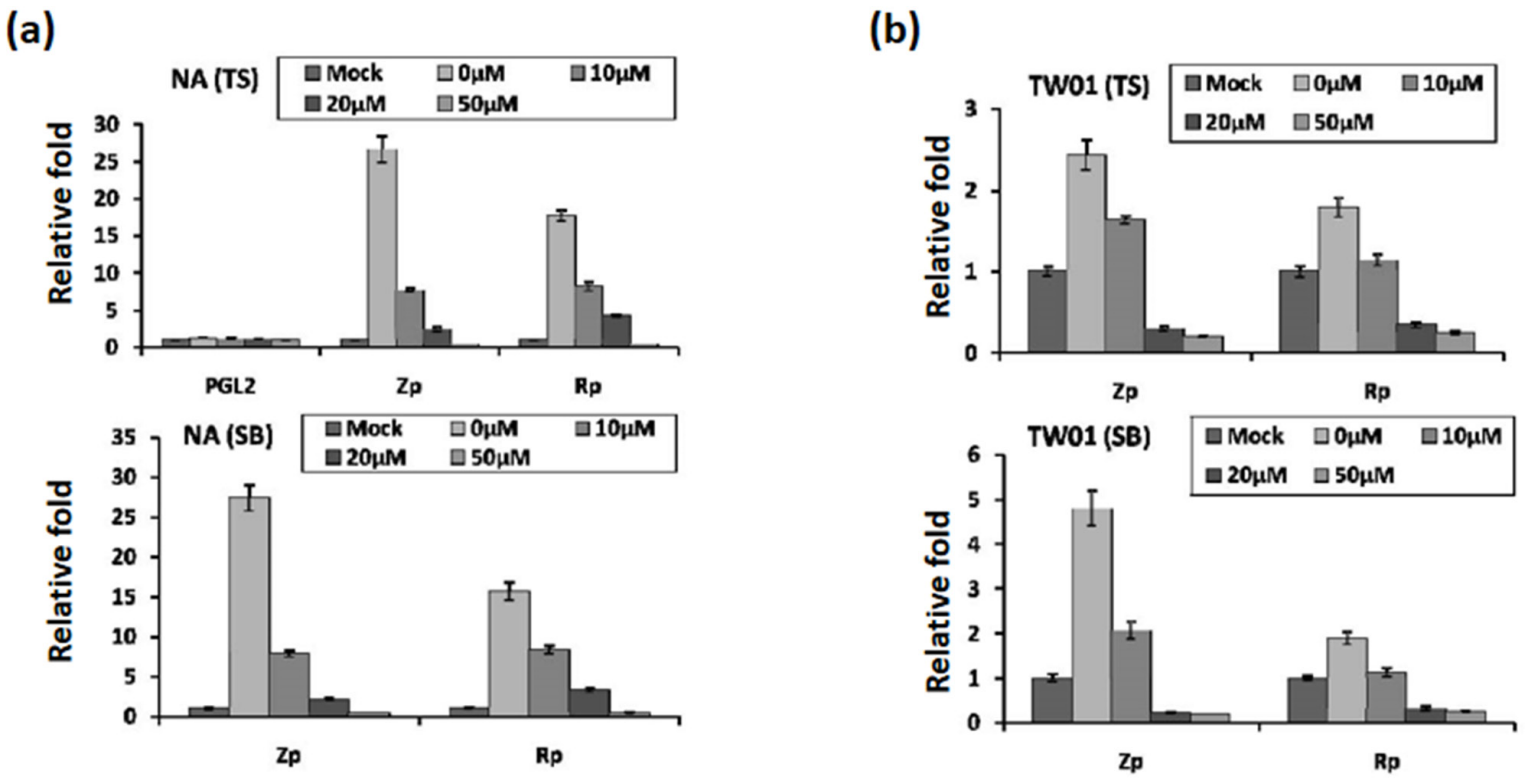
Luteolin represses Zp and Rp activities stimulated by chemical inducers Control plasmid PGL2, Zp, or Rp was transfected into NA cells **a.** and the parental cell line TW01 **b.** After 3∼4 hr of transfection, luteolin was added or not for pre-treatment for 1 hr, and then different methods (TS(TPA+SB) and SB only) were used to induce EBV into the lytic cycle. After induction for 24 hr, cells lysates were collected for measurement of luciferase activity. The mean and standard deviation of each sample was calculated based on duplicates from at least two independent experiments.

Because the activities of the Zta and Rta promoters (Zp and Rp) are crucial for initiation of the EBV lytic cycle, we sought to determine whether luteolin can inhibit their transcriptional activities using a transient transfection assay. NA cells were transfected with Zp (pZp-Luc) or Rp (pRp-Luc) firefly luciferase reporter plasmid for 3 hr. Subsequently, the cells were treated with various amounts of luteolin for 1 hr followed by TS induction for 24 hr. Luciferase activity was subsequently determined as described in Material and Methods. As expected, for the positive control, the luciferase activities of Zp and Rp both increased over 15-fold after TS induction, compared to the mock-transfected control (Figure 4a, upper panel, 0 μM). At the same time, the luciferase activities of Zp and Rp were gradually repressed by increasing concentrations of luteolin (Figure 4a, upper panel). Co-treatment with TS and 50 μM luteolin reduced the activities of Zp and Rp to the mock-transfected control level, meanwhile, transfection of NA cells with the empty vector PGL2 showed that all values were at background levels (Figure 4a, upper panel).

Furthermore, we used SB alone to induce EBV reactivation and tried to analyze this phenomenon in detail. For SB induction, the result was that SB-induced Zp and Rp activities were similar to induction by TS (Figure 4a, lower panel, 0 μM). Even so, luteolin repressed Zp and Rp activities gradually with increasing concentrations (Figure 4a, lower panel, 0-50 μM), compared to the mock control, following SB induction.

To avoid the influence of endogenous EBV in NA cells, we used the parental TW01 cells for the analysis, as described above. The relative folds of Zp and Rp activities were lower than those in NA cells following TS induction (Figure 4b). Similarly, regardless of the treatment (TS or SB), luciferase activities of Zp and Rp were repressed gradually by increasing concentrations of luteolin (Figure 4b), suggesting that luteolin can inhibit Zp and Rp activities following chemical induction.

### Inhibition of recurrent EBV reactivation by luteolin decreased reactivation-induced genomic instability in NA cells

**Figure 5:**
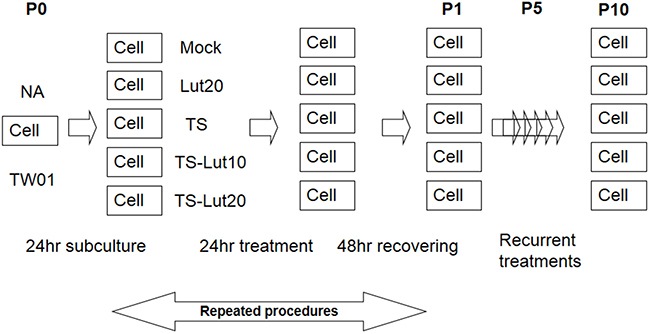
Representative illustration of recurrent chemical treatment of NPC cells Cells were mock or treated repeatedly with the chemicals indicated. The cells at the beginning of this procedure were defined as passage 0 cells (P0). After seeding, the cells were treated with luteolin for 1 hr or not, followed by treatment with TPA (40 ng/ml) and SB (3 mM). After incubation for 24 hr, the cells were allowed to recover by incubation with fresh medium for a further 48 hr. These resulting cells were defined as passage 1 cells (P1). The procedure was repeated for 10 times. “Pn” represents treated NPC cells, where n means the passage number of the cells. TS, Lut10 and Lut20 indicate cells treated with TPA+SB, luteolin 10 μM and luteolin 20 μM.

**Figure 6:**
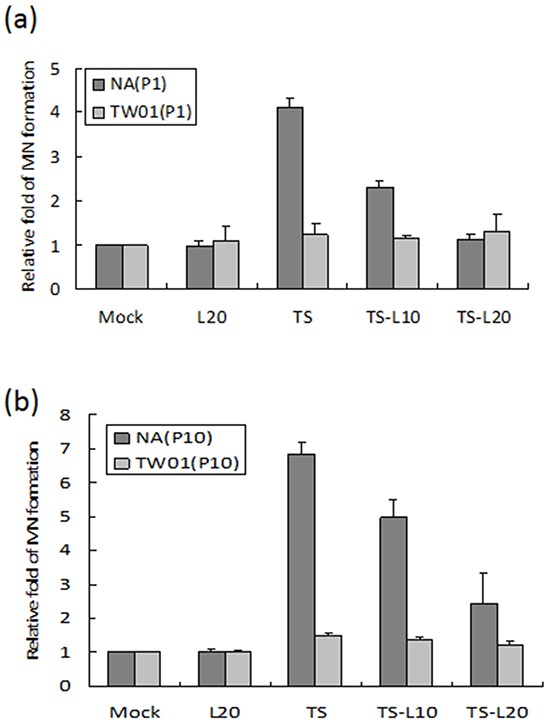
Luteolin inhibits reactivation-induced MN formation The EBV-positive cell line NA and its parental EBV-negative cell line, TW01, with different times of chemical treatment were included in parallel to compare the effects of formation of MN. Cells after **a.** one passage (P1) and **b.** ten passages (P10) treatments were harvested and stained with Hoechst 33258 for MN examination using fluorescence microscope. In all results, the values are a mean±SD from at least three separate experiments. L20: luteolin 20 μM; TS: TPA+SB; TS-L10: TPA+SB+luteolin (10 μM); TS-L20: TPA+SB+luteolin (20 μM).

EBV reactivation has been shown to be important for tumorigenesis. In our previous study, we showed that recurrent EBV reactivation leads to more profound genomic instability and tumorigenesis than seen during EBV latency, implying that EBV reactivation may be a potential target against tumorigenesis [38, 56]. Based on these observations, we postulated that inhibition of the EBV lytic reactivation by luteolin could repress reactivation-induced genomic instability and malignancy. To test this hypothesis, we modified the culture system to carry out repeated reactivation combined with luteolin administration to examine the impact of inhibition of EBV reactivation on genomic instability and the malignant characteristics of NA cells (Figure 5). The EBV-positive cell line NA and its parental EBV-negative cell line TW01 were included in parallel to compare the effects between treatment with inducers (TS) alone (in TW01 cells) and chemical-induced EBV reactivation (in NA cells). MN formation was determined for the alteration of genomic instability. As shown in Figure 6a, TW01 cells after one passage (TW01-P1) with 20 μl luteolin treatment did not increase the extent of MN formation. If the cells in the TW01-P1 group were treated with TS, slightly increased formation of MN was observed; however, no significant difference could be detected in the extent of MN formation under TS and luteolin co-treatment (Figure 6a). On the other hand, in EBV-positive NA cells after one passage (NA-P1), although luteolin alone did not inhibit the formation of MN, it repressed TS-induced MN formation in a dose-dependent manner (Figure 6a). In the TW01 cells after 10 passages (TW01-P10), similar to the TW01-P1 group, low levels of MN formation occurred, regardless of the types of chemical treatment (Figure 6b). This result indicated that, without the impact of EBV, a subtle increase in MN formation was induced in NPC cells following TS treatment, which was similar to our previous observations [38, 56]. Moreover, NA cells after 10 passages with TS treatment (NA-P10) exhibited a more profound increase than the NA-P1 group in terms of the formation of MN, revealing that recurrent EBV reactivation caused accumulation of MN, compared to the mock control (Figure 6b). Furthermore, increasing amounts of luteolin could repress the formation of MN gradually, in both the NA-P1 and NA-P10 groups (Figure 6b). Similarly, luteolin inhibited MN formation after TS induction in C666-1 cells and HA cells (Supplementary Figures S1b and S1d). These results suggest that luteolin can effectively repress the formation of MN induced by EBV reactivation.

### Luteolin inhibits cell proliferation induced by recurrent EBV reactivation

**Figure 7:**
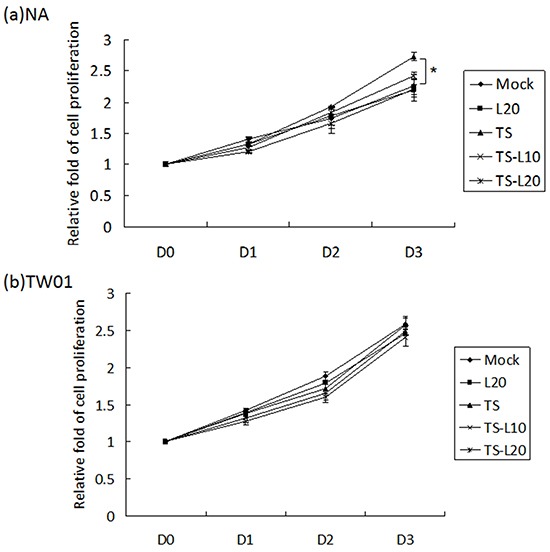
Luteolin represses reactivation-induced cell proliferation **a.** NA and **b.** TW01 cells under repeated treatment were subjected to WST-1 assay to detect the tendency for cell proliferation. In all results, the values are a mean±SD from at least three separate experiments. *: p<0.05. L20: luteolin 20 μM; TS: TPA+SB; TS-L10: TPA+SB+luteolin (10 μM); TS-L20: TPA+SB+luteolin (20 μM).

An increase in cell proliferation is a common feature of carcinogenic cells. To determine whether luteolin can suppress cell proliferation triggered by recurrent EBV reactivation, a WST-1 assay was performed to detect the survival rate of NA and TW01 cells treated by repeated TS induction, without or with luteolin. For NA cells, the TS treatment group exhibited accelerated cell proliferation compared to the mock control; however, this decreased gradually with luteolin treatment (Figure 7a). On the other hand, there was no obvious change among the groups of TW01 cells (Figure 7b). These results suggested that luteolin can inhibit cell proliferation induced by repeated EBV reactivation.

### The tumorigenic properties of NPC cells, induced by EBV reactivation, were repressed with luteolin treatment

**Figure 8:**
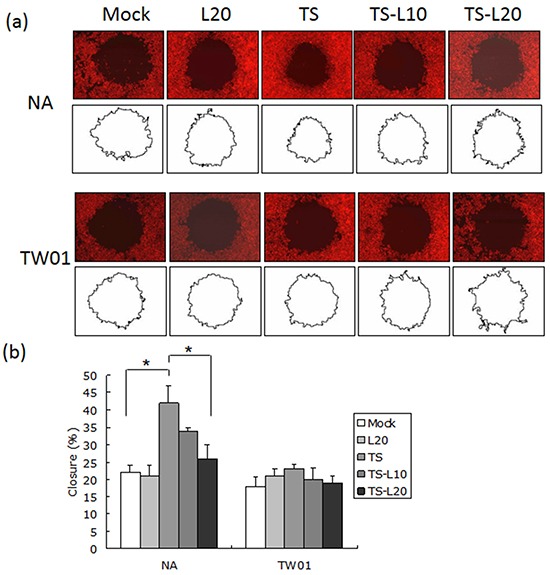
Luteolin represses reactivation-induced cell migration **a.** NA and TW01 cells under repeated treatment were subjected to a cell migration assay, according to the protocol described in Material and Methods. The area of the cell-free zone was measured by Image J software. **b.** Cell migration was determined as percent closure and calculated as described in Materials and Methods. In all results, the values are a mean±SD from at least three separate experiments. *: p<0.05. L20: luteolin 20 μM; TS: TPA+SB; TS-L10: TPA+SB+luteolin (10 μM); TS-L20: TPA+SB+luteolin (20 μM).

**Figure 9:**
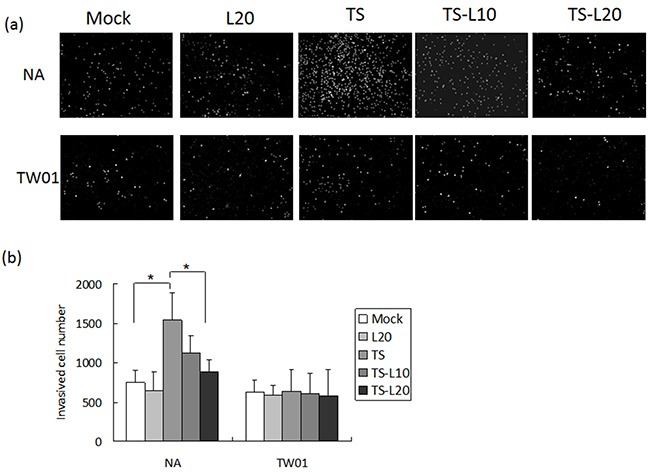
Luteolin represses reactivation-induced cell invasion **a.** NA and **b.** TW01 cells under repeated treatment were subjected to a cell invasion assay, according to the protocol described in Material and Methods. In all results, the values are a mean±SD from at least three separate experiments. *: p<0.05. L20: luteolin 20 μM; TS: TPA+SB; TS-L10: TPA+SB+luteolin (10 μM); TS-L20: TPA+SB+luteolin (20 μM).

In addition to genomic instability, EBV reactivation increased a number of tumorigenic properties in NPC cells, including cell migration, cell invasion and spheroid formation [57]. To determine whether luteolin could inhibit these malignant properties of NPC cells, repeated EBV reactivation was carried out without and with luteolin treatment for analysis of the tumorigenic phenotypes, including cell migration, cell invasion and spheroid formation. EBV-positive NA cells under recurrent TS induction were subjected to cell migration assays and the degrees of migration increased significantly compared to the mock control, however, this decreased following treatment with luteolin (Figure 8a and 8b). This migration inhibition was not seen in TW01 cells treated with luteolin (Figure 8a and 8b). In addition, invasion of NA cells was accelerated in the group undergoing repeated TS induction and this was repressed when luteolin was added (Figure 9a and 9b). This inhibition of invasion also was not seen using TW01 cells treated with luteolin (Figure 9a and 9b).

**Figure 10:**
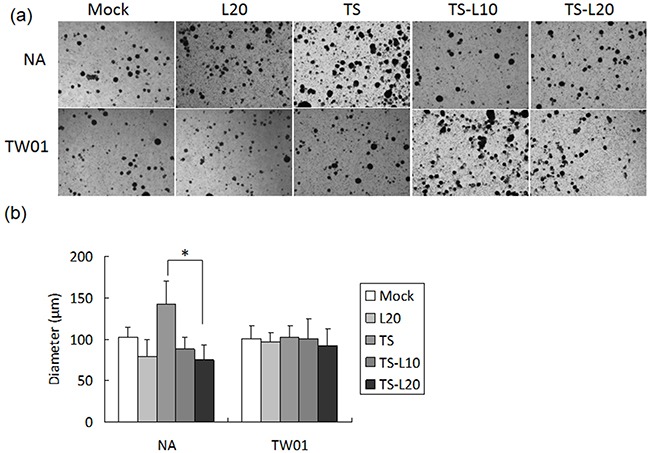
Luteolin represses reactivation-induced spheroid formation **a.** NA and TW01 cells under repeated treatment were subjected to a spheroid assay, according to the protocol described in Material and Methods. **b.** The diameter of the spheroids was measured from images captured by microscopy. In all results, the values are a mean±SD from at least three separate experiments. *: p<0.05. L20: luteolin 20 μM; TS: TPA+SB; TS-L10: TPA+SB+luteolin (10 μM); TS-L20: TPA+SB+luteolin (20 μM).

Cancer cells are present in three-dimensional structures *in vivo*. The formation of multicellular spheroids mimics a tissue-like architecture and exhibits cell-cell contact with intercellular adhesion. Here, in our spheroid formation assay, the TS-treated NA cells formed larger spheroids than the mock control but spheroid formation was repressed after addition of luteolin. However, a similar phenomenon could not be demonstrated in TW01 cells (Figure 10a and 10b). Interestingly, little inhibition of migration and invasiveness was detectable in NA and TW01 cells treated with luteolin alone but this effect was not statically significant (Figures 8 and 9).

### Inhibition of EBV reactivation by luteolin represses tumor growth in a mouse model

**Figure 11:**
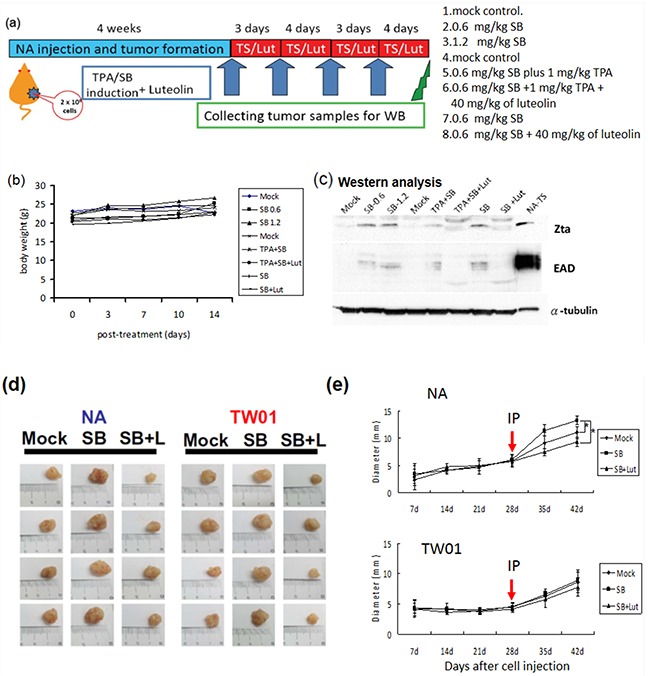
Inhibition of EBV reactivation by luteolin represses tumor growth in mouse model NA cells were inoculated subcutaneously into SCID mice, which then received various treatments. **a.** Representative schedule of EBV reactivation inhibited by luteolin **b.** The record of average animal body weights during the experiment (*n* = 3 mice for each group) (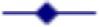 Mock: water injection; 
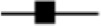
 SB0.6: 0.6 mg/kg SB; 
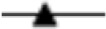
 SB1.2: 1.2 mg/kg SB; 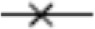 Mock: water injection; 
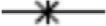
 TPA+SB: 1 mg/kg TPA+ 0.6 mg/kg SB, 
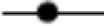
 TPA+SB+Lut: 1 mg/kg TPA+ 0.6 mg/kg SB plus 40 mg/kg luteolin; 
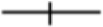
 SB: 0.6 mg/kg SB; 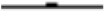 SB+Lut: 0.6 mg/kg SB plus 40 mg/kg luteolin) **c.** The expression of EBV lytic proteins in the tumors excised from the treated mice was analyzed by western blotting. **d.** Sacrificed mice and tumor nodules after excision were photographed at week 2. Mock: water injection; SB: 0.6 mg/kg of sodium butyrate injection; SB+L: 0.6 mg/kg SB plus 40 mg/kg luteolin. **e.** The tumor diameter was measured weekly using callipers. Data are presented as mean±SD. (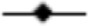 Mock: water injection; 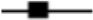 SB: 0.6 mg/kg of SB injection; 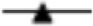 SB+Lut: 0.6 mg/kg SB plus 40 mg/kg luteolin) *: p<0.05

Based on the observation that treatment with luteolin dramatically decreased the tumorigenic properties of NPC cells, induced by recurrent EBV reactivation, we sought to determine whether luteolin repressed tumor growth induced by repeated EBV reactivation in a mouse model (Figure 11a). We first tried to establish the mouse model of EBV reactivation with chemical induction in NA cells. NA cells (2 × 10^6^ cells) were inoculated subcutaneously into SCID mice. After tumor appearance for 4 weeks, the mice were separated into several groups and SB, SB+luteolin, TS and TS+luteolin were administered intraperitoneally every 3 or 4 days (Figure 11a). The tumors were harvested after two further weeks of development and analyzed for Zta and EAD expression to detect EBV reactivation. With no obvious toxicity in terms of mouse body weights (Figure 11b), the tumors from SB and TS–treated mice had significant expression of EBV lytic proteins; however, protein expression was repressed in the luteolin-treated mice (Figure 11c). After successful establishment of the mouse model of EBV reactivation, tumorigenicity experiments were carried out to determine whether luteolin could repress reactivation–induced tumor growth. NA and TW01 cells (2 × 10^6^ cells) were inoculated subcutaneously into SCID mice. After tumor appearance, mice implanted with NA and TW01 cells were separated into several groups and were injected intraperitoneally with mock-treatment (water control), SB or SB+luteolin every 3 or 4 days, as described above (Figure 11a). The tumors were harvested after two further weeks of development for analysis of their growth. In NA-injected mice, we found tumor growth was enhanced after SB administration; however, this was inhibited by luteolin treatment (Figure 11d, left panel and 11e, upper panel). In addition, tumor growth in the SB-treated group was increased with NA cells, but not TW01 cells, at 28-day after SB treatment, compared to the mock control, suggesting EBV reactivation plays a causative role in the tumorigenisis of NPC cells *in vivo* (Figure 11e). Collectively, these results indicate that luteolin has a suppressive effect on NPC tumorigenesis in the mouse model by inhibition of EBV reactivation.

## DISCUSSION

EBV has been associated with many human malignancies, although how it contributes to carcinogenesis is largely unknown [8]. The contribution of the lytic genes of EBV has been the most extensively studied in the etiology of EBV-induced posttransplant lymphoproliferative disorders (EBV-PTLD) after solid organ transplantation (reviewed in [58]). In the clinical course, a monoclonal tumor may develop after polyclonal B-cell proliferation [59]. The association of high rate malignant tumor occurrence with immunosuppression in renal homotransplantation was reported by TE Starzl after observation from 1962 to 1964 [60]. Subsequent reports corroborated this finding [61, 62]. Elevation of antibodies against EBV was found in patient sera [63] and EBV was detected in the oropharyngeal secretions [64]. Further studies indicated the disease may also be caused by EBV reinfection [65] or primary infection [66]. Treatment with acyclovir suppressed polyclonal B-cell proliferation [67] and PTLD [68], indicating that EBV reactivation plays an important role in these disorders. It was suggested that cytotoxic T cells against EBV-infected B cells were suppressed in renal transplant recipients after immunosuppression [69]. Withdrawal of immunosuppression was found to render the regression of PTLD, supporting the crucial role of cellular immunity in the development of PTLD [70]. With the development of EBV replication-defective mutants, in which the immediate early genes BZLF1 and BRLF1 were knocked out [71], it was possible to study the contribution of EBV lytic genes to the oncogenesis of PTLD. BZLF1 was shown to be important in the development of LCL tumors in SCID mice through induction of vascular endothelial growth factor (VEGF) [72]. BZLF1 also was found to enhance the tumor formation by BZLF1-deleted LCL in SCID mice, through increased expression of IL-6 and IL-10 [73]. A humanized mouse model containing human CD34 cells (hematopoietic cells), thymus and liver tissues provided evidence that host cellular immunity contributes to the suppression of development of LPD [74]. Using superlytic (SL) mutants and OKT3 and anti-CD3 antibodies, a further study supported the contributions of EBV lytic infection and human cellular immunity to the development of LPC [75]. It was suggested that horizontal transmission of EBV may be important for PTLD formation and lytic EBV may contribute through paracrine effects and/or immunosuppression [74]. Similarly, reactivation of EBV was found to contribute to the tumorigenesis of NPC cells [38]. In this study, we wanted to determine whether reactivation of EBV can be a target in the management of NPC after remission.

For the contribution of lytic EBV genes to NPC tumorigenesis, we found chemical induced reactivation of EBV can promote genome instability and tumor formation in SCID mice [38, 56, 76]. In this study, we screened out the flavonoid luteolin as a viral inhibitor to block latent EBV entry into the lytic cycle. We demonstrated that appropriate doses of luteolin can inhibit the expression of EBV lytic proteins through repression of Zp and Rp activities (Figures 2-4), suggesting that luteolin is a promising agent for inhibiting the initial stage of latent EBV transition to lytic reactivation. Furthermore, we also found that luteolin treatment blocked the formation of MN and several tumorigenic properties of NPC cells (Figures 7-10). In a newly established mouse model, luteolin repressed reactivation-induced tumor formation (Figure 11), suggesting that luteolin can repress NPC tumorigenesis by inhibiting EBV reactivation.

“Antimicrobial adjuvant therapy” has been proposed recently to treat virus-related cancers and cancer-associated infections. Targeting cancer-related viruses with antiviral agents is currently used in the treatment of hepatocellular carcinomas [77], HHV8-associated cancers [78] and hematopoietic cancers [79], and this has yielded some compelling data to support their clinical use in combination with traditional therapies. Several anti-EBV or induction-lytic strategies have been proposed for the treatment of EBV-related malignancies [80–91]. Based on our previous studies, the EBV lytic cycle was found to be crucial to the tumorigenesis of NPC cells and some lytic proteins are known to have mutagenic or carcinogenic properties [38–41]. Taking EBV reactivation as an oncotarget, it is worth considering retarding or repressing the recurrence of NPC using natural products.

Luteolin is a flavonoid found in our diet, albeit in relatively low amounts (<1 mg/day) [50, 52]. Epidemiological studies have revealed an inverse correlation between luteolin consumption and the risk of some cancers [92–95]. Furthermore, luteolin has been shown to have significant anti-carcinogenic effects through induction of anti-oxidation, anti-proliferation and apoptosis via multiple signaling pathways, *in vitro* and *in vivo* [52, 96]. Luteolin has been shown to block Akt phosphorylation in TNF-α induced murine non-carcinoma intestinal epithelial cells [97]. Luteolin also has inhibitory effects in MAPK/ERK signaling and the PI3-K cascade [98, 99]. In our laboratory, we found luteolin can repress phosphorylation of the ERK, p38, JNK and PKC pathways in NA cells (data not shown). Of note, blocking of the Akt, MAPK/ERK or PI3K pathways was shown to be important in preventing EBV reactivation [100, 101], suggesting inhibition of these signals by luteolin may contribute to its anti-viral and anti-cancer properties.

In investigating its modes of action, luteolin was found to cause significant inhibition of Zp and Rp activities (Figure 4). The mechanism is similar to EGCG and andrographolide, which also inhibit Zp and Rp activities [102, 103]. On the contrary, curcumin and retinoid acid inhibit Zp [104] and moronic acid inhibits Rp activity [105]. In addition, SFN inhibits EBV reactivation by interfering with Rta transactivity [106]. Because Zp activation is the most important step in EBV reactivation in B cells, whereas Rp activation is critical in epithelial cells, compounds inhibiting both promoter activities, e.g. luteolin and EGCG, have better potential for clinical application. In addition, we found that luteolin blocks EBV reactivation by repressing the Zta and Rta promoter activities, disrupting Sp1 binding (data not shown). These functions will provide more potential for anti-cancer and anti-viral therapy.

In summary, we show that luteolin inhibits EBV reactivation by repressing the promoter activities of Zp and Rp. Furthermore, through inhibition of EBV reactivation, luteolin decreases viral reactivation-induced genomic instability and malignant features, such as cell proliferation, migration, invasion and spheroid formation. Luteolin also represses tumor growth in a mouse model. We propose here that EBV reactivation may be a novel target for prevention or retardation of NPC relapse and luteolin may provide a new solution to the drug-resistance problem and have potential as a lead for drug development. It also provides an alternative choice for antiviral therapy and prevention.

## MATERIALS AND METHODS

### Compounds and antibodies

Luteolin and the induction agents, 12-O-tetradecanoyl-phorbol-1,3-acetate (TPA) and sodium butyrate(SB), were purchased from Sigma-Aldrich Co. Antibodies used in this study include anti-EBV Rta 467 (unpublished), anti-BMRF1 (EAD) 88A9 [107], anti-EBV Zta 4F10, anti-DNase 311H [108], anti-β-actin (Sigma-Aldrich Co.) and anti-GAPDH (Sigma-Aldrich Co.).

### Cell lines

TW01 is a human EBV-negative NPC cell line established from a Taiwanese NPC patient [36]. HONE-1 is another human EBV-negative NPC cell line, from a Chinese NPC patient [109]. NA and HA cells, are EBV converted cells obtained by co-culture of rAkata cells and TW01 and HONE-1 cells, respectively, and were selected by G418 (Sigma-Aldrich Co) treatment [35]. All of the above cell lines and their derivatives were maintained in DMEM (Dulbecco's modified Eagle's medium) supplemented with 10% fetal calf serum (FCS). C666-1 is a NPC cell lines derived from an NPC xenograft of southern Chinese origin [110]. It is maintained in RPMI-1640 supplemented with 5% FCS.

### EBV induction by TPA plus SB and luteolin administration

The EBV-positive NPC cell lines (NA and HA) were seeded for 24 hr before carrying out the experiments. To determine whether luteolin can inhibit EBV reactivation, the cells were pre-treated for 1 hr with various concentrations of luteolin. TPA (40 ng/ml) and SB (3 mM) were added subsequently to co-treat to the cells for EBV induction. After 24 or 48 hr incubation, the cells and their extracts were collected for further studies.

### Flow cytometric analysis

To determine the number of cells which switched into the lytic cycle, cells were treated as indicated, harvested and fixed in 70% ethanol. The fixed cells were permeabilized with 1% Triton X-100 and 4% FBS and incubated with anti-EAD antibody (dilution 1:10) for 2 hr at room temperature. The cells were washed with PBS and incubated with a 1:1000 dilution of goat anti-mouse IgG rhodamine-conjugated antibody for 1 hr and then washed and analyzed using a Becton Dickinson FACScan flow cytometer (BD Biosciences, San Jose, CA). Each experiment was in duplicate with 10,000 cells.

### Transfection and analysis of luciferase reporter activity

The construction of the Zp and Rp reporter plasmids has been described in previous reports [76, 106, 111]. Zp and Rp reporter plasmids were transfected using Lipofectamine 2000 (Invitrogen), according to the manufacturer's instructions. Briefly, NA or TW01 cells were seeded (2×10^5^/well) for Zp and Rp activation by TPA/SB. The Zp or Rp plasmid, mixed with Lipofectamine 2000 (Invitrogen) in Opti-MEM medium (Invitrogen), was incubated for 20 min, then added to the culture wells containing the cells. After 3 hr transfection, luteolin was added or not for pre-treatment for 1 hr, and then TPA (40 ng/ml) plus SB (3 mM) or SB (3 mM) alone were added to induce EBV into the lytic cycle. After induction for 24 hr, the cells were lysed in 50 μl HEPES buffer (0.1M HEPES, pH 7.8, 1% Triton X-100, 1 mM CaCl_2_ and 1 mM MgCl_2_) and 25 μl of the lysates were combined with 25 μl of Luciferase Assay Reagent II (Promega) for 10 min incubation. Finally, the luciferase activity was measured using a luminescence counter (Packard). Each lysate sample was quantified for the expression of β-actin to control for variation in the amount of sample (data not shown). The mean and standard deviation of each sample were calculated from three independent experiments in duplicate.

### Determination of MN formation

Detection of MN formation was carried out as described previously [24]. The cells under repeated treatment with inducers combined with or without luteolin were seeded onto coverslips to adhere. After 24 h incubation, the culture medium was removed and the cells were washed twice with phosphate-buffered saline (PBS, pH7.4). The cells were fixed with ice-cold methanol for 15 min. After washing twice with PBS, the cells were stained with Hoechst. (0.2 μg/ml, Sigma-Aldrich, St. Louis, MO) for 15 minutes. Micronuclei were judged using a fluorescence microscope.

### Cell proliferation assay

The cytotoxicity of luteolin to each cell line was determined by WST-1 assay (Invitrogen) according to manufacturer's instructions. Briefly, NA and TW01 cells (5×10^3^ cells/well) and HA and HONE-1 cells (1×10^4^ cells/well) were cultured in 96 well plates for 24 hr and 48 hr. Various concentrations of luteolin (0, 1, 5, 10, 20, 50 and 100 μM) were incubated with the cells for 24 or 48 hr and the cytotoxicity was analyzed by WST-1 assay. The fluorescence was measured with a microplate reader (Infinite M200, Tecan). The half maximum of cytotoxicity concentration (CC_50_) was defined as the concentration of luteolin which killed 50% of the cells. The results of at least three independent experiments were used to calculate the mean and standard deviation.

### Cell migration assay

Cell migration assays were carried out using the Oris system, according to the manufacturer's instructions (Platypus Technologies). Briefly, the cells were seeded in 96-well plates with Oris stoppers and then incubated for 24 hr. The stoppers were then removed and the cells were incubated for a further 24 hr to permit cell migration. Finally, the cells were analyzed with PI staining and photographed by microscopy (Olympus). The closure of the cell-free zone was detected by Image J software (National Institute of Health). The cell migration was presented as the percentage of closure calculated using the following equation: [(pre-migration)_area_-(migration)_area_/(pre-migration)_area_]x100. The results of at least three independent experiments were used to calculate the mean and standard deviation.

### *In vivo* tumorigenesis model

Six-week-old SCID mice were inoculated subcutaneously with NA cells (2 × 10^6^ cells) and examined over 4 weeks for tumor appearance. When the tumor size reached approximately 0.5 cm in diameter, the mice were separated into groups, each containing three mice. The 1^st^ group served as the control. The 2^nd^, 3^rd^ and 4^th^ groups received 0.6, 1.2 mg/kg SB and 0.6 mg/kg SB plus 1 mg/kg TPA, respectively, delivered by IP injection every 3 or 4 days. The 5^th^ and 6^th^ groups received 40 mg/kg of luteolin, delivered by IP injection every 3 or 4 days, before treatment with the inducers. Two weeks later, the animals were sacrificed and the tumors were excised and extracted to examine the expressions of EBV lytic proteins. For *in vivo* tumorigenesis assays, mice were inoculated subcutaneously with NA cells for 4 weeks and, when the tumor size reached approximately 0.5 cm in diameter, the mice were separated into three groups, each containing six mice. The 1^st^ group served as the control, the 2^nd^ group received 0.6 mg/kg SB, delivered by IP injection every 3 or 4 days, the 3^rd^ group received 40 mg/kg of luteolin, delivered by IP injection every 3 or 4 days, before the treatment with the inducers. The health of the mice and tumor sizes were monitored 3 or 4 days and the diameters of tumors were measured using calipers. Two weeks later, the animals were sacrificed and the tumors were excised for measurement and weighing.

### Spheroid assay

Cells were seeded into non-coating 10 cm-plates to maintain their suspension and incubated at 37°C for 7 days. After incubation, the spheroids sizes were photographed and their sizes determined using Image J software.

## SUPPLEMENTARY FIGURE


