# Supplementary material for: EBV reactivation as a target of luteolin to repress NPC tumorigenesis
Source: Oncotarget. 2016 Mar 8;7(14):18999–9017. doi: 10.18632/oncotarget.7967 (PMC4951347; doi:10.18632/oncotarget.7967)
Supplement: Supplementary file 1 [file oncotarget-07-18999-s001.pdf]

SUPPLEMENTARY FIGURES

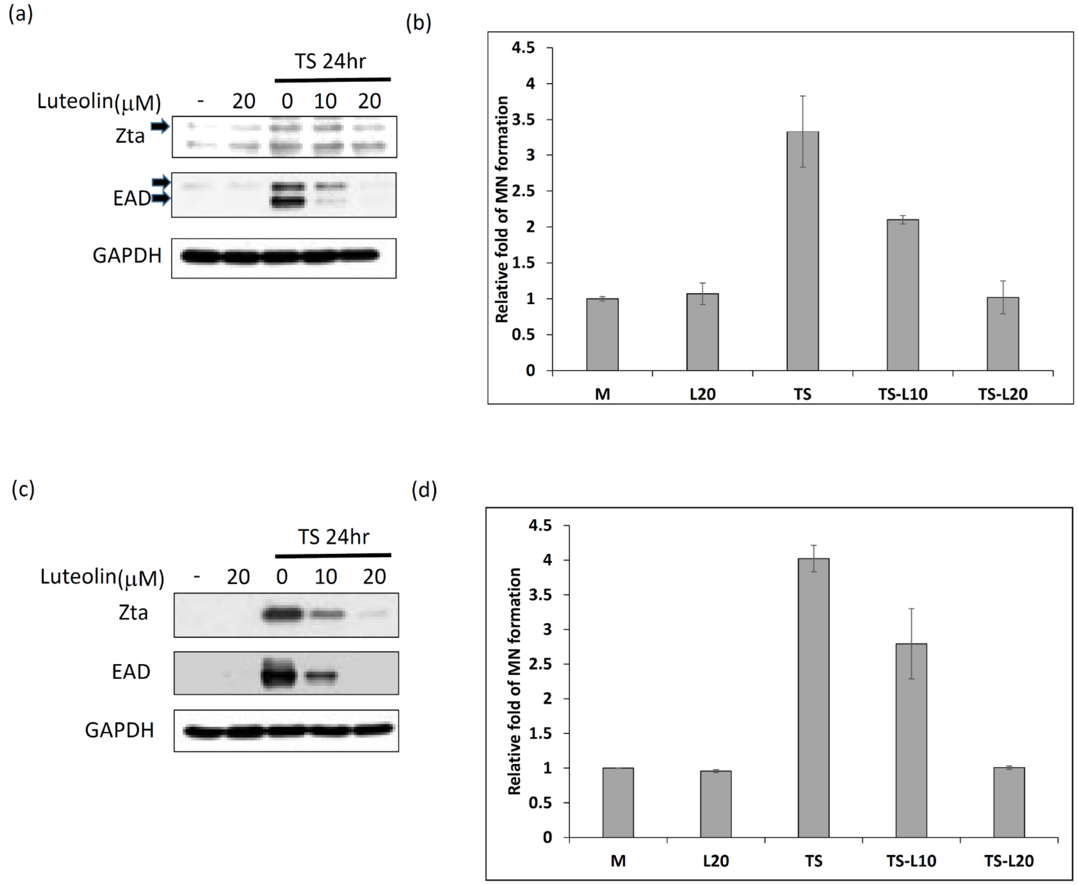

Supplementary Figure 1: Luteolin inhibits EBV reactivation and MN formation in C666-1 and HA cells. C666-1 [1] and HA epithelial cells were subjected to western blotting and MN formation. **a.** C666-1 cells were pre-treated with arious concentrations of luteolin for 1 hr, then TPA (40 ng/ml) and SB (3 mM) co-treatment was used to induce EBV reactivation. After 24 hr of incubation, cell lysates were analyzed by western blotting with antibodies against EBV Zta [2], EAD [3] and GAPDH (Sigma-Aldrich Co.). **b.** For detection of MN formation, C666-1 cells were collected and stained with Hoechst 33258 (Sigma-Aldrich Co.) and examined by fluorescence microscopy. **c.** HA cells were treated using the procedure described above. The lysates were examined by western blotting using the same antibodies against EBV Zta, EAD and GAPDH and **d.** the cells were collected and stained with Hoechst 33258 for detection of MN formation. In all cases, the values are the mean±SD from three individual experiments.

REFERENCES

1. Cheung ST, Huang DP, Hui AB, Lo KW, Ko CW, Tsang YS, Wong N, Whitney BM and Lee JC. Nasopharyngeal carcinoma cell line (C666-1) consistently harbouring Epstein-Barr virus. Int J Cancer. 1999; 83:121-126.
2. Tsai CH, Liu MT, Chen MR, Lu J, Yang HL, Chen JY and Yang CS. Characterization of monoclonal antibodies to the Zta and DNase proteins of Epstein-Barr virus. J Biomed Sci. 1997; 4:69-77.
3. Tsai CH, Williams MV and Glaser R. Characterization of two monoclonal antibodies to Epstein-Barr virus diffuse early antigen which react to two different epitopes and have different biological function. J Virol Methods. 1991; 33:47-52.
